# Robot-assisted reconstruction using ureteroureterostomy and Lich–Gregoir ureteral reimplantation for complicated duplex kidneys in children

**DOI:** 10.1371/journal.pone.0351941

**Published:** 2026-06-17

**Authors:** Huazhang Liu, Chang Tao, Xiang Yan, Guangjie Chen, Guangping Zeng

**Affiliations:** 1 Department of Urology, Children’s Hospital, Zhejiang University School Of Medicine, National Clinical Research Center For Children And Adolescents’ Health And Diseases, Hangzhou, Zhejiang, PR China; 2 Department of Neonatology, Hangzhou Women’s Hospital, Hangzhou obstetrics and Gynecology Hospital Affiliated to Hangzhou Normal University, Hangzhou, Zhejiang, PR China; West Bengal University of Animal and Fishery Sciences, INDIA

## Abstract

**Objective:**

This study aimed to evaluate the efficacy of robot-assisted reconstruction using end-to-side ureteroureterostomy (UU) and Lich‒Gregoir ureterovesical reimplantation (UR) for complicated duplex kidneys in children.

**Methods:**

A retrospective study was conducted on pediatric patients who underwent combined robotic UU and Lich‒Gregoir UR between January 1, 2021, and January 1, 2024. The inclusion criterion was the presence of concurrent upper- and lower-pole ureteral pathologies. Surgical time, postoperative length of stay, pre- and postoperative anteroposterior diameter of the renal pelvis (APD), ureteral diameter (UD), renal function (RF) of the affected kidney, and complications were analyzed.

**Results:**

In total, 12 patients presented with urinary tract infections (UTI, n = 9), incontinence (n = 2), and abdominal pain (n = 1). Upper-pole pathologies included ectopic ureters (10 cases) and ureteroceles (2 cases). Lower-pole pathologies comprised ureteral stricture (n = 4) and vesicoureteral reflux (VUR, n = 8). The median surgical time was 177.5 minutes (range: 140–205 minutes), and the median hospital stay was 5 days (range: 3–7 days). Postoperative complications included two cases of UTI, both managed conservatively. At a median follow-up of 19 months (range: 12–31 months), no anastomotic stricture, urinary leakage, or ureteral stump syndrome occurred. Postoperative voiding cystourethrogram (VCUG) revealed no VUR in any patient. Pre- and postoperative APD (22.92 ± 9.07 mm vs. 7.33 ± 5.03 mm, p < 0.001) and UD (11.08 ± 3.15 vs. 5.83 ± 2.41 mm, p < 0.001) differed significantly in the upper pole system, as did the lower pole APD (14.00 ± 6.25 vs. 5.25 ± 3.02 mm, p < 0.001) and UD (7.75 ± 2.26 vs. 4.75 ± 1.36 mm, p = 0.003). The RF of the affected moiety improved significantly (36.58 ± 4.66% vs. 42.75% ± 3.10%, p < 0.001).

**Conclusions:**

Robot-assisted reconstruction using UU and Lich–Gregoir UR is a safe and effective approach for complex duplex kidneys requiring concurrent upper- and lower-tract reconstruction, demonstrating durable resolution of obstruction and reflux as well as functional preservation.

## Introduction

Duplex kidneys, characterized by complete or incomplete duplication of the collection system, are among the most prevalent congenital urinary tract anomalies in the pediatric population, with an estimated incidence of 0.5% to 0.8% of live births. The majority of cases involve pathological lesions in a single upper pole ureter, such as an ectopic ureter or ureterocele, which can be managed by ureteral reimplantation or ureteroureterostomy [1]. A subset of patients (approximately 10%–15%) present with complicated cases involving concurrent ipsilateral lower-pole ureteral abnormalities, including high-grade VUR (grades IV–V) or ureterovesical junction obstruction (UVJO).

These complex presentations pose unique surgical challenges. Traditional open approaches require extensive dissection and are associated with increased risks of neurovascular injury, whereas conventional laparoscopy is limited by restricted instrument maneuverability within the confined pediatric pelvic space. With the advent of robotic technology, surgeons have been able to overcome many of the limitations inherent to conventional laparoscopy. Robotic technology offers enhanced precision, improved visualization, and the ability to perform delicate procedures with minimal invasiveness, thus providing more favorable outcomes for pediatric patients [2,3]. However, the current literature predominantly focuses on isolated UU or UR procedures; the feasibility and outcomes of combined robotic UU and UR for duplex systems with dual pathologies remain underexplored.

In this study, we present the first dedicated evaluation of a novel robot-assisted approach that combines end-to-side UU with Lich‒Gregoir UR for the treatment of duplicated renal anomalies. We hypothesized that this combined technique would lead to improved surgical outcomes, including a lower complication rate and better long-term preservation of renal function. By evaluating the safety and efficacy of this approach, we seek to provide valuable insights into its potential as a preferred treatment modality for complex cases of duplex kidneys.

## Materials and methods

This study enrolled consecutive pediatric patients who underwent robot-assisted reconstruction using UU and Lich–Gregoir UR between January 1, 2021, and January 1, 2024, at a tertiary pediatric urology center (Department of Urology, Children’s Hospital, Zhejiang University School of Medicine). Diagnostic confirmation of duplex kidneys with ipsilateral upper- and lower-pole ureteropathies was achieved through standardized imaging methods: renal ultrasonography, VCUG, and magnetic resonance urography (MRU). Pathological findings included upper-pole ectopic ureters or ureteroceles with coexisting lower-pole pathologies, such as ureteral stricture or VUR.

Preoperative and postoperative renal functional assessments of the affected renal unit were performed using 99mTc-DTPA renal scintigraphy. Owing to the presence of lesions in both the upper and lower ureters on the same side, we calculated the RF for the entire affected kidney (both upper and lower moieties) before and after surgery. Indications for the procedure were as follows: 1) duplicated kidneys with concurrent ipsilateral upper- and lower-pole ureteropathies requiring dual reconstruction; 2) progressive hydronephrosis of the affected moiety due to obstruction; 3) symptomatic presentations, including refractory urinary incontinence, obstructive abdominal pain, or recurrent urinary tract infections (UTIs); and 4) preserved differential renal function (≥10% of total renal function) in the affected moiety on preoperative 99mTc-DTPA scintigraphy. The contraindications were as follows: 1) nonsalvageable renal moieties with aplastic development or advanced functional impairment (differential renal function < 5%) and 2) active pyonephrosis with systemic sepsis or unresolved parenchymal infection in the affected moiety.

This study was approved by the Ethics Committee of Children’s Hospital, Zhejiang University School of Medicine (2024-IRB-0227-P-01). As this study exclusively involved retrospective analysis of preexisting anonymized medical data without implementing any patient interventions, the ethics committee waived the requirement for additional informed consent in accordance with institutional ethical review regulations. Research data extraction was systematically conducted from February 1, 2025, to February 15, 2025. All medical records underwent rigorous de-identification prior to analysis: 1) automated removal of the 18 HIPAA-defined identifiers by our hospital’s medical record management system and 2) manual verification by independent staff to ensure non-linkability. We confirm that all methods were performed in accordance with the relevant guidelines.

### Robotic platform and system configuration

All robot-assisted procedures were performed using the fourth-generation da Vinci Xi Surgical System (Intuitive Surgical, Sunnyvale, CA, USA). The system was equipped with a high-definition stereoscopic 3D imaging system with automatic multi-angle view switching, enabling approximately 10 × magnification of the surgical field. Four 8-mm robotic arms provided approximately 540° of multidirectional rotation, offering sufficient maneuverability for precise manipulation under complex anatomical conditions. In addition, an ergonomically designed console effectively filtered physiologic hand tremors and, combined with wristed instruments providing seven degrees of freedom, enhanced the stability and accuracy of intracorporeal fine surgical maneuvers.

### Surgical procedure

**Fig 1:**
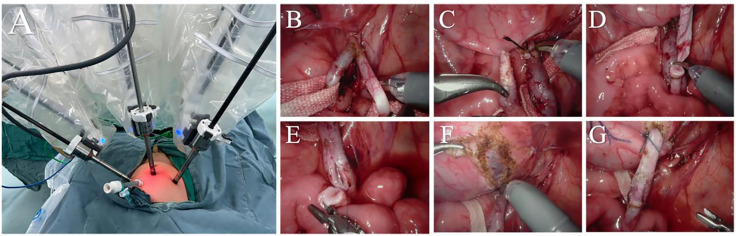
Illustration of end-to-side ureteroureterostomy coupled with Lich‒Gregoir ureteral reimplantation. **(A)** Port placement. **(B)** Exposure of both the upper and lower ureters. **(C)** Dissection of the upper ureter. **(D)** Resection of the redundant upper ureter. **(E)** Ureteral tailoring and preparation for anastomosis. **(F)** Construction of the submucosal tunnel. **(G)** Closure of the muscle layer of the posterior bladder wall.

After the induction of general anesthesia, the patient was placed in the supine position. An 8-mm camera was introduced through the umbilicus. Following the establishment of pneumoperitoneum, two 8-mm robotic trocars were placed bilaterally near the umbilicus, and a 5-mm auxiliary trocar was inserted between the left robotic trocar and the instrument trocar. The distance between the ports was maintained at greater than 6 cm (Fig 1A).

The peritoneum was incised at the level of the iliac vessels to expose and identify the upper and lower ureters (Fig 1B). The upper ureter was fully mobilized and transected above the ureteral common sheath (Fig 1C). The distal ureteral stump was ligated, and the redundant portion of the upper ureter was excised (Fig 1D). A longitudinal incision corresponding in width to the transected ureter was made on the lateral wall of the lower ureter (Fig 1E). The anastomosis site was typically located more than 5 cm from the distal end of the lower ureter, ensuring an adequate length for ureteral reimplantation. End-to-side anastomosis was performed using a continuous 6–0 absorbable suture.

The bladder was suspended with a 3−0 suture and filled with 0.9% saline via a urine catheter to maintain a straight bladder wall. In cases of VUR affecting the lower pole ureter, cystoscopy was performed prior to the robotic surgery, and a double-J stent was inserted into the lower pole ureter. There was no need to detach the lower pole ureter. Afterward, the lateral muscle layer of the posterior bladder wall was dissected to establish a submucosal tunnel, maintaining a length-to-ureteral diameter ratio of 5:1 (Fig 1F). In cases of lower ureteral stricture, the ureter was transected at the ureterovesical junction, and the strictured segment was excised. An incision was made in the bladder mucosa below the tunnel, and a double-J stent was inserted percutaneously. Afterward, the lower ureter was anastomosed to the newly created ureteral orifice with a 6−0 suture, followed by the formation of a submucosal tunnel. The muscle layer of the rear bladder wall was closed with a 4–0 absorbable suture (Fig 1G).

### Data collection and follow-up

The following parameters were recorded: operative time, postoperative hospital stay, postoperative complications, anteroposterior diameter of the APD, UD, and RF of the affected kidney. Patients received anti-inflammatory therapy postoperatively. The drainage strips were removed once the drainage volume was less than 5 mL. The patients were discharged after catheter removal, provided that they exhibited no symptoms of UTI, abdominal pain, or hematuria. The double-J stent was removed 4–6 weeks postoperatively. Follow-up ultrasounds were performed every 3 months postoperatively, and VCUG and renal scans were conducted at 6 months postoperatively. Surgical success was defined as the resolution of symptoms or a reduction or complete resolution of hydronephrosis on ultrasound.

### Statistical analysis

Statistical analysis was conducted using SPSS version 26.0, with statistical significance set at P < 0.05. Continuous variables following a normal distribution were presented as the mean and standard deviation (X ± s). Non-normally distributed data were expressed as the median and interquartile range (IQR). Preoperative and postoperative APD, UD, and RF were compared using paired t-tests.

## Results

During the study period, 12 patients, including 4 males and 8 females, underwent this robot-assisted procedure. The procedure was performed on the left side in 8 patients and on the right side in 4 patients. The patients’ ages ranged from 6 to 135 months, and the median age was 13 months. Nine patients presented with UTIs, 2 with incontinence, and 1 with abdominal pain. Pathological findings in the upper pole system included ectopic ureters (10 cases) and ureteroceles (2 cases), while the lower pole system exhibited ureteral stricture (4 cases) and VUR (8 cases). Additionally, 2 patients had VUR in the contralateral renal system, and contralateral ureteral reimplantation was performed in one of them. Patient demographics were summarized in Table 1.

**Table 1:** Patient demographics and perioperative data.

Age (months), median (range)	13	(6-135)
**Gender, n (%)**		
Male	4	33.3%
Female	8	66.7%
**Laterality, n (%)**		
Lt	8	66.7%
Rt	4	33.3%
**Type of pathology, n (%)**		
**Upper pole system**		
Ureteral ectopia	10	83.3%
Ureterocele	2	16.7%
**Lower pole system**		
Ureteral stricture	4	33.3%
VUR	8	66.7%
**Contralateral renal system, n (%)**		
VUR	2	16.7%
**Surgical indication，n (%)**		
Urinary incontinence	2	16.7%
Febrile UTI	9	75%
Abdominal pain	1	8.3%
**Operative time (minutes), median (range)**	177.5	140-205
**Tube drainage stay (days), median (range)**	2	1-4
**Hospital stay (days), median (range)**	5	3-7
**Complications, n (%)**		
UTI	2	16.7%

Abbreviations: n, number; Lt; left; Rt; right; VUR, vesicoureteral reflux; UTI, urinary tract infection.

All surgeries were successfully completed without conversion to open surgery. The operative time ranged from 140 to 205 minutes (median: 177.5 minutes). The postoperative hospital stay varied from 3 to 7 days (median: 5 days). Pelvic drainage tubes were removed between 1 and 4 days postoperatively (median: 2 days). Two patients developed postoperative UTIs one week after double-J stent removal, both of which were resolved with conservative antibiotic therapy. No cases of urinary leakage, anastomotic stricture, or ureteral stump syndrome were observed.

The follow-up period ranged from 12 to 31 months (median 19 months). Postoperative VCUG confirmed the absence of VUR in all patients. There were statistically significant differences in the pre- and postoperative APD and UD for both the upper pole system (22.92 ± 9.07 mm vs. 7.33 ± 5.03 mm, p < 0.001; 11.08 ± 3.15 vs. 5.83 ± 2.41 mm, p < 0.001) and the lower pole system (14.00 ± 6.25 mm vs. 5.25 ± 3.02 mm, *p* < 0.001; 7.75 ± 2.26 mm vs. 4.75 ± 1.36 mm, p = 0.003). The renal function of the affected kidney significantly improved (36.58% ± 4.66% vs. 42.75% ± 3.10%, p < 0.001). Preoperative and postoperative data are summarized in Table 2.

**Table 2:** Preoperative and postoperative data.

	Preoperative	Postoperative	*P* value
**Upper pole system**			
APD (mm）	22.92 ± 9.07	7.33 ± 5.03	**<0.001**
UD (mm)	11.08 ± 3.15	5.83 ± 2.41	**<0.001**
**Lower pole system**			
APD (mm）	14.00 ± 6.25	5.25 ± 3.02	**<0.001**
UD (mm)	7.75 ± 2.26	4.75 ± 1.36	**0.003**
**RF (%)**	36.58 ± 4.66	42.75 ± 3.10	**<0.001**

Abbreviations: APD, anteroposterior diameter of the renal pelvis; UD, ureteral diameter; RF, renal function.

## Discussion

According to the Weigert–Meyer law, the ureter from the upper renal moiety in a duplicated kidney often inserts ectopically and inferiorly, which can lead to continuous urinary dribbling in female patients and the formation of a ureterocele, potentially resulting in urinary obstruction and recurrent urinary tract infections. Conversely, the ureter from the lower renal moiety typically inserts more directly and with a shorter course into the bladder, which makes it more prone to VUR. In addition, ureterovesical junction strictures are commonly associated with the lower pole ureter. Owing to the significant individual variations in the location of ectopic ureteral openings, the mechanisms underlying VUR, and the presence of ureteral strictures, the clinical management of this condition remains highly complex and has become a major challenge in pediatric urology [4].

In recent years, treatment strategies for duplicated kidneys have focused primarily on preserving the function of the affected kidney. Additionally, an increasing number of studies have reported the use of a single UU or UR for managing pediatric duplicated kidneys [5–8]. Laparoscopic techniques provide several advantages, including faster recovery, shorter hospital stays, and fewer complications, which make them a preferred option for treating duplex kidney malformations in pediatric patients. Tao et al. analyzed 30 children with complete renal duplication, of whom 20 underwent laparoscopic ureteroureterostomy (LUU) and 10 underwent open ureteroureterostomy (OUU). Their findings indicated that, compared with open surgery, laparoscopic procedures resulted in less surgical trauma, faster postoperative recovery, and superior postoperative recovery of anatomical parameters (APD, UD) of the upper kidneys [9]. Similarly, Zhu et al. investigated 90 children with duplicated kidneys who underwent Lich‒Gregoir UR, including 35 who underwent open surgery and 55 who underwent laparoscopy. The authors reported that laparoscopic Lich‒Gregoir UR was successful, clinically effective, and safe for a pediatric population with functional duplex kidneys, with outcomes superior to those of open surgery techniques [10]. Additionally, on comparing the efficacy of two techniques in pediatric patients with complete renal duplication, Yu et al. found that both procedures were safe and effective [11].

However, laparoscopic surgery is generally associated with a longer learning curve, and surgeons may encounter technical challenges when managing complex reconstructions. At present, studies specifically addressing the learning curve of UU and UR remain limited. In this context, data from pediatric pyeloplasty provide valuable insight into the relative learning characteristics of laparoscopic and robotic approaches. In a multi-institutional study of infants with ureteropelvic junction obstruction (UPJO), learning curve analysis based on operative time demonstrated that laparoscopic pyeloplasty (LP) plateaued after approximately 18 cases, whereas robot-assisted laparoscopic pyeloplasty (RALP) plateaued after approximately 13 cases, suggesting a shorter early learning phase for the robotic approach. Notably, the RALP continued to show a second phase of improvement after approximately 37 cases, reflecting ongoing refinement with increasing experience [12]. Similarly, Zhou et al. used cumulative sum (CUSUM) analysis to characterize the learning curve of pediatric RALP, demonstrating that competency was achieved by the 11th case. Collectively, these findings suggest that robotic platforms may facilitate earlier technical stabilization in pediatric reconstructive surgery [13]. However, continued experience remains essential to achieve optimal efficiency and procedural consistency.

Early clinical experience with robot-assisted ureteral reconstruction was mainly accumulated on the da Vinci Si and earlier platforms. Rodriguez et al. retrospectively analyzed a single-center series of patients with duplex kidneys and VUR who underwent robot-assisted extravesical common-sheath ureteral reimplantation, and reported a radiographic success rate of 87.50% with no high-grade complications [14]. Esposito et al. in a systematic review including 22 studies, summarized outcomes in 1,362 children treated with robot-assisted ureteral reimplantation (RALUR) and reported an overall patient success rate of 92%, a mean postoperative complication rate of 10.7%, and a mean reoperation rate of 3.9% [15]. In contrast, all cases in the present study were performed using the da Vinci Xi system, with similarly satisfactory outcomes. Compared with da Vinci Si and earlier platforms, the Xi system may offer certain platform-related advantages in the confined operative field of pediatric surgery, mainly in terms of arm positioning, port configuration, and docking workflow. For example, its overhead boom design may improve flexibility in arm positioning, its slimmer arms with additional joints may facilitate closer port spacing and reduce external arm collisions, and laser-guided docking with integrated targeting may improve docking efficiency. However, these advantages are primarily technical and workflow-related, and whether they result in improved clinical outcomes requires further study [16].

The applications of the da Vinci Xi system in pediatric urology mainly include RALP for UPJO, RALUR for UVJO or VUR, robot-assisted partial nephrectomy (RALPN) and robot-assisted ureteroureterostomy (RALUU) for duplex kidney anomalies, as well as other upper urinary tract reconstructive procedures in children [16–18]. Beyond pediatric urology, the Xi system has also been introduced in pediatric general and neonatal surgery (e.g., fundoplication, esophageal atresia, and diaphragmatic hernia repair), pediatric thoracic surgery (e.g., thymic and mediastinal procedures), and selected pediatric oncologic surgeries such as renal tumor resection, supporting its applicability to complex pediatric surgery [19–21]. Notably, these procedures share key technical features with UU and UR, including meticulous dissection with the preservation of tissue vascularity and the need for precise intracorporeal suturing within a confined operative field.

Ipsilateral ureteral anomalies involving both ureters are uncommon, with clinical manifestations primarily including recurrent febrile urinary tract infections (fUTIs) and varying degrees of renal function deterioration. Historically, common-sheath UR has been the primary treatment method [22,23]. However, the procedure typically requires a wider and longer submucosal tunnel, which can lead to a mismatch between tunnel length and ureter diameter. This mismatch increases the risk of VUR, particularly in infants and young children with smaller bladders. In our cohort, the median surgical age was 13 months. With respect to this younger age group, performing common-sheath ureteral reimplantation presents substantial technical challenges. Lee et al. reported a postoperative VUR incidence of 5%–10% and a reoperation rate of 17.9% after common-sheath reimplantation [24]. Rodriguez et al. conducted a clinical study involving 13 children with duplicated kidneys who underwent robot-assisted common-sheath ureteral reimplantation and reported a postoperative VUR rate of 12.5% [14].

Our findings suggest that the combination of UU and UR effectively addresses the discrepancy in the tunnel-to-ureteral diameter ratio, thereby reducing the incidence of postoperative VUR. Robot-assisted surgery provides significant advantages in terms of ureteral dissection, anastomosis, and submucosal tunnel creation. High-resolution 3D visualization and flexible robotic arms allow for precise ureteral dissection with minimal tissue damage. In ureteral anastomosis, enhanced dexterity and wristed instrumentation enable meticulous suturing with reduced tension, lowering the risk of stricture and urinary leakage. Additionally, the creation of a robot-assisted submucosal tunnel ensures an optimal length-to-diameter ratio, minimizing the incidence of VUR while preserving bladder integrity.

The design of port placement directly determines the adequacy of the operating space for robot-assisted surgery. In our study, the port placement strategy was similar to that used in RALUR. For patients older than 1 year, the camera was placed at the umbilical margin, and two operation ports were placed on both sides of the horizontal line. For patients under 1 year old, the camera port was placed 1–2 cm above the umbilicus to ensure sufficient operating space for robot-assisted surgery.

In this study, all 12 patients (aged 6–135 months) underwent successful surgeries without conversion to open surgery, and our cohort also showed a shorter hospital stay than that reported in previously published laparoscopic series of complex duplex kidney surgery (mean hospital stay, 11.20 ± 2.60 days) [25]. However, given the descriptive nature of the present case series and the absence of a contemporaneous comparator cohort, this difference should be interpreted as an observational finding rather than as direct evidence that the technical advantages of robotic surgery improve postoperative recovery.

UU can be performed at high levels (inferior pole of the kidney) or low levels (pelvic level) [26,27]. We prefer performing anastomosis at the pelvic level, as this region is more superficial, facilitating a technically simpler procedure. This approach minimizes extensive mobilization of the upper ureter, thereby reducing the risk of lower ureteral injury. UR includes intravesical Cohen and Politano–Leadbetter procedures, as well as the extravesical Lich–Gregoir approach. Robot-assisted intravesical UR poses challenges in infants with a small bladder, whereas the Lich‒Gregoir technique is more feasible, as it avoids bladder incision and preserves bladder mucosal integrity. Additionally, this approach is associated with shorter durations of hematuria and catheterization, as well as a lower incidence of postoperative bladder spasms [15,28].

To prevent ureteral stricture and urinary leakage due to excessive mobilization, we opted to preserve a wider ureter and construct a longer submucosal tunnel. Intraoperatively, the length of the bladder mucosal tunnel was increased by obliquely incising the bladder detrusor muscle. Follow-up examination revealed that none of the 12 pediatric patients in this study developed VUR or anastomotic stenosis. Considering the risk of ureteral stump infection, our study emphasizes the importance of completely excising the distal ureter segment and ligating the ureteral stump, particularly in patients with tortuous and dilated ureters or those with associated VUR. Robot-assisted surgeries several advantages, including enhanced visualization, greater flexibility, and more precise mechanical arms, enabling thorough excision of the ureteral stump. Notably, no ureteral stump infections occurred in this study, suggesting that robotic surgery may lead to a lower incidence of ureteral stump syndrome.

## Conclusions

Robot-assisted reconstruction using UU and Lich–Gregoir UR is a safe and feasible approach for complex duplex kidneys requiring concurrent upper- and lower-tract reconstruction. During follow-up, hydronephrosis and ureteral dilation were reduced, renal function improved, and the overall complication rate remained low. However, given the descriptive nature of this case series, the absence of a contemporaneous comparator cohort, and the small sample size, these findings should be interpreted with caution and require further validation in larger prospective controlled studies.

## Supporting information

S1 File PLOSOne Human Subjects Research Checklist. (PDF)

S2 File Minimal data set for this study. (XLSX)
